# Complete Genome Sequences for 59 *Burkholderia* Isolates, Both Pathogenic and Near Neighbor

**DOI:** 10.1128/genomeA.00159-15

**Published:** 2015-04-30

**Authors:** Shannon L. Johnson, Kimberly A. Bishop-Lilly, Jason T. Ladner, Hajnalka E. Daligault, Karen W. Davenport, James Jaissle, Kenneth G. Frey, Galina I. Koroleva, David C. Bruce, Susan R. Coyne, Stacey M. Broomall, Po-E Li, Hazuki Teshima, Henry S. Gibbons, Gustavo F. Palacios, C. Nicole Rosenzweig, Cassie L. Redden, Yan Xu, Timothy D. Minogue, Patrick S. Chain

**Affiliations:** a Los Alamos National Laboratory (LANL), Los Alamos, New Mexico, USA; b United States Army Medical Research Institute of Infectious Diseases (USAMRIID) Diagnostic Systems Division (DSD), Fort Detrick, Maryland, USA; c Naval Medical Research Center (NMRC)-Frederick, Fort Detrick, Maryland, USA; d Henry M. Jackson Foundation, Bethesda, Maryland, USA; e United States Army Medical Research Institute of Infectious Diseases (USAMRIID) Center for Genome Sciences (CGS), Fort Detrick, Maryland, USA; f United States Army Edgewood Chemical Biological Center (ECBC), Aberdeen Proving Ground, Aberdeen, Maryland, USA

## Abstract

The genus *Burkholderia* encompasses both pathogenic (including *Burkholderia mallei* and *Burkholderia pseudomallei*, U.S. Centers for Disease Control and Prevention Category B listed), and nonpathogenic Gram-negative bacilli. Here we present full genome sequences for a panel of 59 *Burkholderia* strains, selected to aid in detection assay development.

## GENOME ANNOUNCEMENT

*Burkholderia mallei* and *Burkholderia pseudomallei* are among the bacterial species considered to be potential bioweapons, along with *Bacillus anthracis*, *Brucella melitensis*, *Brucella abortus*, and *Yersinia pestis* (1, 2). *B. pseudomallei* causes melioidosis, often a respiratory infection mimicking tuberculosis, while *B. mallei* generally infects horses, causing glanders. The listing of these bacteria as potential biothreats is due to their easy availability (*B. pseudomallei* is often recovered from soils in regions where it is endemic), their ability to cause severe and often fatal disease, multiple routes of infection, native antibiotic resistance, lack of available vaccines, wide host range, and ability to persist in the environment for weeks to years (3–9). *B. mallei* was reportedly used as a biological weapon on several occasions (10–14); however, while *B. pseudomallei* was investigated for its use as a bioweapon, there are no reports that it has been employed in this fashion (5, 11). Other *Burkholderia* species are opportunistic pathogens (e.g., the *Burkholderia cepacia* complex [Bcc] that adversely affects cystic fibrosis patents [including 7 species sequenced here]), plant pathogens (such as *Burkholderia gladioli*) and/or common soil microorganisms. Here we present full genome sequences of 59 strains useful for detection assay development, including both species that should be detected (inclusivity) and those that should not be (exclusivity).

Draft genome assemblies included two or more data sets (specific data types and coverages are listed in the NCBI records): Illumina (short- and/or long-insert paired data), Roche 454 (long-insert paired data), and PacBio long reads. Short- and long-insert paired data were assembled together in both Newbler and Velvet, and computationally shredded into 1.5-kbp overlapping shreds. If the PacBio coverage was 100× or greater, the data were assembled using PacBio’s Hierarchical Genome Assembly Process (HGAP) (15). All data were additionally assembled together in Allpaths whenever possible (16). Consensus sequences from both HGAP and Allpaths were computationally shredded into 10-kbp overlapping pieces. All shreds were integrated using Phrap. Possible misassemblies were corrected and repeat regions verified using in-house scripts and manual editing in Consed (17–19). All of the genomes were assembled into finished-quality complete genomes (20). Each genome assembly was annotated using an Ergatis-based (21) workflow with minor manual curation.

Genome assemblies range from 5.4 to 9.7 Mb (Table 1, mean 6.96 ± 0.014 Mb), with two or three chromosomes and up to three plasmids. As expected for the genus, the G+C content was high, averaging 67.7%.

**TABLE 1:** Listing of *Burkholderia* isolate genomes released to NCBI

Species and isolate	Accession no. (no. of contigs)^*a*^	Panel^*b*^	Genome (bp)	No. of plasmids	No. of CDSs^*c*^	G+C content (%)
*B. ambifaria*						
AMMD	CP009797–CP009800	E	7,528,578	1	6,602	67
*B. cepacia*						
LMG 16656	JTDP00000000 (5)	E	7,923,342	1	7,278	68
*B. dolosa*						
AU0158	CP009793–CP009795	E	6,409,095	2	5,657	67
*B. fungorum*						
ATCC BAA-463	CP010024–CP010027	E	9,058,983	1	8,206	62
*B. gladioli*						
ATCC 10248	CP009319–CP009322	E	8,899,459	3	7,561	68
*B. glumae*						
ATCC 33617	CP009432–CP009435	E	6,820,727	2	5,864	68
*B. mallei*						
6	CP008710–CP008711	I	5,647,769	0	4,872	68
11	CP009587–CP009588	I	5,913,134	0	5,083	68
NCTC 10247	CP007801–CP007802	I	5,827,656	0	5,001	68
2000031063	CP008731–CP008732	I	5,874,930	0	5,067	68
2002721276	CP010065–CP010066	I	5,780,439	0	4,954	69
2002734299	CP009337–CP009338	I	5,740,115	0	4,966	68
2002734306	CP009707–CP009708	I	5,409,162	0	4,703	68
China5	JPNX00000000 (2)	I	5,869,855	0	5,043	68
FMH 23344	CP008704–CP008705	I	5,625,292	0	4,883	68
India86-567-2	CP009642–CP009643	I	5,686,446	0	4,911	68
KC_1092	CP009942–CP009943	I	5,661,851	0	4,868	68
*B. multivorans*						
BAA-247	CP009830–CP009832	E	6,322,746	0	5,607	67
*B. oklahomensis*						
C6786	CP009555–CP009556	E	7,135,022	0	6,083	67
EO147	CP008726–CP008727	E	7,313,673	0	6,312	67
*B. pseudomallei*						
9	CP008753–CP008755	I	7,228,737	1	5,978	68
576	CP008777–CP008778	I	7,266,604	0	5,944	68
1026b	CP004379–CP004380	I	7,450,511	0	6,113	68
1106a	CP008758–CP008759	I	7,086,433	0	5,758	68
7894	CP009535–CP009536	I	7,381,912	0	6,036	68
PB08298010	CP009550–CP009551	I	7,375,551	0	6,023	68
K96243	CP009537–CP009538	I	7,247,614	0	5,933	68
MSHR 146	CP004042–CP004043	I	7,313,103	0	5,963	68
MSHR 1655	CP008779–CP008780	I	7,027,950	0	5,798	68
MSHR 2543	CP009477–CP009478	I	7,446,569	0	6,183	68
MSHR 305	CP006469–CP006470	I	7,428,072	0	6,105	68
MSHR 346	CP008763–CP008764	I	7,354,416	0	6,015	68
MSHR 406e	CP009297–CP009298	I	7,271,506	0	5,927	68
MSHR 491	CP009484–CP009485	I	7,356,376	0	6,080	68
MSHR 511	CP004023–CP004024	I	7,316,085	0	5,964	68
MSHR 520	CP004368–CP004369	I	7,447,511	0	6,113	68
MSHR 668	CP009545–CP009546	I	7,042,714	0	5,793	68
MSHR 840	CP009473–CP009474	I	7,129,813	0	5,860	68
NAU 20B-16	CP004003–CP004004	I	7,313,851	0	5,969	68
NAU 35A-3	CP004377–CP004378	I	7,204,083	0	5,844	68
NCTC 13178	CP004001–CP004002	I	7,408,007	0	6,133	68
NCTC 13179	CP003976–CP003977	I	7,337,157	0	6,085	68
Pasteur 52237	CP009898–CP009899	I	7,325,318	0	6,015	68
PHLS 112	CP009585–CP009586	I	7,202,363	0	5,868	68
*B. thailandensis*						
2002721643	CP009601–CP009602	E	6,722,801	0	5,649	68
2002721687	CP009547–CP009549	E	7,285,824	1	6,327	67
2002721723	CP004097–CP004098	E	6,577,133	0	5,533	68
2003015869	CP008914–CP008915	E	6,728,980	0	5,679	68
34	CP010016–CP010018	E	7,120,198	1	6,129	67
E254	CP004381–CP004382	E	6,676,730	0	5,591	68
E264	CP008785–CP008786	E	6,722,099	0	5,655	68
E444	CP004117–CP004118	E	6,651,696	0	5,571	68
H0587	CP004089–CP004090	E	6,768,375	0	5,629	68
Malaysia 20	CP004383–CP004384	E	6,684,359	0	5,620	68
MSMB 121	CP004095–CP004096	E	6,731,379	0	5,758	68
Phuket 4W-1	AQQJ00000000 (3)	E	6,674,944	0	5,635	68
*B. ubonensis*						
MSMB 22	CP009486–CP009488	E	7,189,071	0	6,257	67
*B. vietnamiensis*						
LMG 10929	CP009629–CP009632	E	6,930,496	1	6,120	67
*B. xenovorans*						
LB400	CP008760–CP008762	E	9,702,951	0	8,684	63

a Contig count is listed only for genomes at Improved High Quality Draft (IHQD) quality; all others are finished (20).

b E, exclusivity strain; I, inclusivity strain.

c CDS, coding sequence.

### Nucleotide sequence accession numbers. 

Accession numbers for all 59 genomes are listed in Table 1.
