# Correction for Johnson et al., Complete Genome Sequences for 59 *Burkholderia* Isolates, Both Pathogenic and Near Neighbor

**DOI:** 10.1128/genomeA.00313-16

**Published:** 2016-04-21

**Authors:** Shannon L. Johnson, Kimberly A. Bishop-Lilly, Jason T. Ladner, Hajnalka E. Daligault, Karen W. Davenport, James Jaissle, Kenneth G. Frey, Galina I. Koroleva, David C. Bruce, Susan R. Coyne, Stacey M. Broomall, Natkunam Ketheesan, Mark Mayo, Alex Hoffmaster, Mindy Glass Elrod, Vanaporn Wuthiekanun, Apichai Tuanyok, Robert Norton, Bart J. Currie, David M. Wagner, Paul Keim, Po-E Li, Hazuki Teshima, Henry S. Gibbons, Gustavo F. Palacios, C. Nicole Rosenzweig, Cassie L. Redden, Yan Xu, Timothy D. Minogue, Patrick S. Chain

**Affiliations:** a Los Alamos National Laboratory (LANL), Los Alamos, New Mexico, USA; b United States Army Medical Research Institute of Infectious Diseases (USAMRIID) Diagnostic Systems Division (DSD), Fort Detrick, Maryland, USA; c Naval Medical Research Center (NMRC)-Frederick, Fort Detrick, Maryland, USA; d Henry M. Jackson Foundation, Bethesda, Maryland, USA; e United States Army Medical Research Institute of Infectious Diseases (USAMRIID) Center for Genome Sciences (CGS), Fort Detrick, Maryland, USA; f United States Army Edgewood Chemical Biological Center (ECBC), Aberdeen Proving Ground, Aberdeen, Maryland, USA; g James Cook University, Townsville, Queensland, Australia; h Menzies School of Health Research, Casuarina, Northern Territory, Australia; i U.S Centers for Disease Control and Prevention, Atlanta, Georgia, USA; j Mahidol University, Bangkok, Thailand; k University of Florida, Gainesville, Florida, USA; l Townsville Hospital, Douglas, Queensland, Australia; m Northern Arizona University, Flagstaff, Arizona, USA

## AUTHOR CORRECTION

Volume 3, no. 2, e00159-15, 2015. Page 1: The byline and affiliation line should read as given above.

Page 3: In Table 1, isolates 2002721687 and MSMB 121 should be identified as *Burkholderia humptydooensis* (proposed).

